# Multiple studies and weak evidential defeat

**DOI:** 10.1007/s11017-017-9409-9

**Published:** 2017-07-04

**Authors:** Nikk Effingham, Malcolm J. Price

**Affiliations:** 1 Department of Philosophy, University of Birmingham, Birmingham, UK; 2 Biostatistics, Evidence Synthesis and Test Evaluation, Institute of Applied Health Research, College of Medical and Dental Sciences, University of Birmingham, Edgbaston, Birmingham, B15 2TT UK

**Keywords:** Philosophy of epidemiology, Multiple testing, Multiplicity, Bayesian, Evidential defeat

## Abstract

When a study shows statistically significant correlation between an exposure and an outcome, the credence of a real connection between the two increases. Should that credence remain the same when it is discovered that further independent studies between the exposure and other independent outcomes were conducted? Matthew Kotzen argues that it should remain the same, even if the results of those further studies are discovered. However, we argue that it can differ dependent upon the results of the studies.

## Introduction

Let φ and ψ be any two factors. A study provides significant results between φ and ψ if and only if that study reports a statistically significant correlation between φ and ψ which would have had only a 1% probability of occurring in the absence of a real connection. A real connection exists between φ and ψ if and only if some variety of causal connection can be traced between φ and ψ. Chance alone allows for studies to show significant results when no real connection exists. Chance alone also allows a real connection to exist between factors without significant results appearing in a study, though, for the sake of argument, this article assumes that this never happens.

Imagine reading a study showing significant results between φ and ψ. It is then discovered that further independent studies examining the connection between φ and some other outcomes have been conducted. ‘Defeatists’ believe that if those studies are discovered, the subjective probability of there being a real connection between φ and ψ (i.e., our rational credence) should necessarily lower. But Matthew Kotzen argues to the contrary [1]. Whilst this article does not contest that the mere existence of the other studies is irrelevant, we argue that Kotzen goes too far in thinking that the results of the studies are also irrelevant.

## Defeatism

Imagine the following:

‘Single study scenario’: We read a study showing significant results between ingesting peanut butter and cholesterol lowering.

Say RealConnection is the proposition that those factors have a real connection (i.e., that eating peanut butter has a causal connection with lower cholesterol). Say SignificantResults is the proposition that a study on peanut butter consumption and lower cholesterol shows significant results. Before reading the study, a person has a measure of belief that peanut butter lowers cholesterol, which presumably goes up once she reads the study, i.e., P(RealConnection|SignificantResults) > P(RealConnection).

Now, imagine that Imaginary Peanut Butter Inc., the fictional commissioning body of the make-believe study, has conducted 999 other studies. Each study examined the association between peanut butter and a factor other than lower cholesterol (e.g., peanut butter and conception rates, peanut butter and resistance to dengue fever, etc.). Call this the ‘multiple study scenario’. Given that the probability of a study producing significant results when there is no real connection is (by our definition of ‘statistically significant’) 1%, on average, roughly ten of the 1000 studies conducted in the multiple study scenario would show significant results by mere chance alone. If, for example, 11 of the studies returned significant results, the likeliest explanation would be that one study reflects a real connection whilst the other ten are mere stochastic detritus. Since the lower cholesterol study is but one study amongst the eleven, it is probable that it is amongst the mere detritus. Therefore, one should think P(RealConnection|SignificantResults) is lower in the multiple study scenario than in the single study scenario. This is the reasoning of the ‘defeatist’.

Defeatists can be divided into two varieties: strong and weak. Strong defeatists believe that in all such situations, the existence of further studies necessarily lowers one’s credence that the original study shows a real connection. Specifically, the strong defeatist believes that the results of those studies are irrelevant. Weak defeatists believe that merely knowing of the existence of further studies does not lower one’s credence of there being a real connection. Instead, the weak defeatist says one needs to know what the results of those studies are. Further, in light of those results, the credences that the connection assessed in the original study is real may remain unchanged, decrease, or increase.

Kotzen argues that both forms of defeatism are false [1]. We do not contest strong defeatism being false, but we do argue that weak defeatism is true.

## Kotzen’s dice analogies

### Single and multiple dice scenarios

Kotzen’s first argument against defeatism depends upon an analogy. Imagine a vat of 1,000,000 dice. One percent of the dice are biased and are perfectly weighted to always roll a ‘6’; the remainder are fair. A die is selected from the whole vat. Call the die ‘Harry’. HarryIsBiased is the proposition that Harry is biased. P(HarryIsBiased) clearly mirrors the percentage of biased dice in the vat, i.e., P(HarryIsBiased) = 0.01. But now imagine that Harry is rolled three times and comes up ‘6’ each time. Call this the ‘single die scenario’.

In the single die scenario, what should the probability be of Harry being biased? That is, where Three6s is the proposition that Harry came up with three 6s in a row, what is P(HarryIsBiased|Three6s)? It is easy to calculate. Given Kolmgorov’s axioms,

$${\text{P}}({\textsc{HarryIsBiased}}|{\textsc{Three6s}}) = \frac{{{\text{P}}({\textsc{Three6s}} {\wedge} {\textsc{HarryIsBiased}})}}{{{\text{P}}({\textsc{Three6s}})}}$$

Obviously, P(Three6s) is equal to P(Three6s∧HarryIsBiased) + P(Three6s∧¬HarryIsBiased). Further, given De Finetti’s axiom,

$${\text{P}}({\textsc{Three6s}} {\wedge}{\neg} {\textsc{HarryIsBiased}}) = {\text{P}}({\textsc{Three6s}}|{\textsc{HarryIsBiased}}) \times {\text{P}}({\textsc{HarryIsBiased}}) = 0.01$$

and

$${\text{P}}({\textsc{Three6s}} {\wedge} {\neg} {\textsc{HarryIsBiased}}) = {\text{P}}({\textsc{Three6s}}|{\neg} {\textsc{HarryIsBiased}}) \times {\text{P}}({\neg} {\textsc{HarryIsBiased}}) \approx 0.005.$$

Overall, P(HarryIsBiased|Three6s) ≈ 0.686.

Now consider a different scenario. Imagine that rather than rolling Harry, Harry is placed back in the vat and then 100,000 dice are randomly selected from the vat and all rolled three times. Of the dice that come up ‘6’ each time, one is randomly selected. What is the probability that such a die is biased? Call this the ‘multiple dice scenario’. Where the die in the single die scenario is called Harry, call the selected die in the multiple dice scenario ‘Laura’. So the question is, what value does P(LauraIsBiased) take?

The answer is straightforward: exactly the same as P(HarryIsBiased|Three6s). Imagine the 100,000 dice are instead rolled one by one. Imagine that the first die to be rolled rolls three 6s in a row. We put it aside and call it ‘Laura’. Clearly, Laura is now just like Harry from the single die scenario, i.e. P(LauraIsBiased) ≈ 0.686. It is equally clear that the rolling of 99,999 more dice is irrelevant to that probability—rolling those dice would not make any difference to the probability of Laura being biased. This scenario, where the dice are rolled one by one, is functionally identical to the multiple dice scenario, for it makes no difference whether the dice are rolled one by one or all together. Further, it makes no difference whether the dice are rolled one by one and Laura happens to be the first die rolled, or whether all the dice are rolled simultaneously and Laura is the one picked out from amongst the dice that rolled three 6s. Kotzen’s conclusion, with which we concur, is that the same credence should be given to Laura and Harry being biased in both scenarios.

On the back of this conclusion, Kotzen argues that defeatism is false because the pairs of scenarios are analogous. The single die scenario is analogous to the single study scenario. Rolling three 6s in a row is analogous to a random study showing significant results, i.e. P(Three6s) is analogous to P(SignificantResults). Randomly selecting a biased die is analogous to randomly selecting a study on factors which bear a real connection to one another, i.e. P(HarryIsBiased) is analogous to P(RealConnection). A die which has rolled three 6s in a row being biased is analogous to a study that shows significant results being on factors bearing a real connection, i.e. P(HarryIsBiased|Three6s) is analogous to P(RealConnection|SignificantResults).

Similarly, the multiple dice scenario is analogous to the multiple study scenario. Multiple dice being rolled is the same as multiple studies being conducted. In the same way that ‘Laura’ is defined as one of the dice that rolled three 6s, the study in the multiple study scenario is guaranteed to be one from amongst those that showed significant results. In the same way that P(HarryIsBiased|Three6s) = P(LauraIsBiased), the probability of a study being on factors with a real connection, given that one is reading a study which shows significant results, is the same in both the single study scenario and the multiple study scenario. That is, the existence of the other studies is irrelevant to one’s credence that the study one is reading is on factors with a real connection or not, i.e. strong defeatism is false.

### The relevant disanalogy

The multiple dice scenario makes no mention of the results of the other 99,999 dice; whatever they rolled is irrelevant to P(LauraIsBiased). If the multiple dice scenario is analogous to the multiple study scenario, then the results of the other studies are irrelevant to P(RealConnection|SignificantResults) and weak defeatism is also false. It is at this step that we find fault since the dice scenarios are subtly disanalogous to the study scenarios. Start by examining one reason Kotzen considers for thinking that P(LauraIsBiased) < P(HarryIsBiased|Three6s). Recall that in the multiple dice scenario Harry is pulled out of the vat but is not rolled. Instead, Harry is replaced and the 100,000 dice are rolled instead (from which Laura is selected). We nevertheless have a value of P(HarryIsBiased), and given the mathematics above, P(LauraIsBiased) is a function of it. If the results of the 100,000 dice affect P(HarryIsBiased) then they would likewise affect P(LauraIsBiased). But Kotzen thinks that the results of the dice rolls should not alter P(HarryIsBiased):

After all, we know that the jar contains a large number of (stipulatively randomized) dice, and we know that 99% of them are fair and that 1% of them are biased. Presumably, even the Defeatist wants to allow that when you pick *just one* die … at random from a jar with such a composition of dice, a rational agent’s prior credence in [HarryIsBiased] should be .01. [1, pp. 163–164]

The first sentence in this quote is the bone of contention. In the dice case, Kotzen *stipulates* that the proportion of biased dice is known. But when studies are conducted, one cannot be certain of the proportion of studies which will be on factors with a real connection. Thus, there is a disanalogy.^1^

We start with the dice scenarios. In the original dice scenario, P(HarryIsBiased) mirrors the percentage of dice believed to be biased (i.e. P(HarryIsBiased) = 0.01) and does not alter when the rest of the dice are rolled. But when one becomes uncertain about the percentage of biased dice, this need not be true. Just as long as the results of the dice influence, to some degree, one’s belief about what proportion of dice are biased, the results of the dice bear on P(HarryIsBiased). Intuitively, this is so. Imagine that it is unclear what proportion of the dice are biased. 100,000 dice are rolled and *all* come up three 6s in a row. One would then strongly suspect that all of the dice, Laura included, are biased, i.e., fix P(HarryIsBiased) close to 1 (and, by extension, P(LauraIsBiased) would be close to 1). But if 100,000 dice are rolled and 50,231 roll three 6s, then that result would be most likely if 50% were biased; if we then fixed P(HarryIsBiased) at 0.5, P(LauraIsBiased)—being the same as P(HarryIsBiased|Three6s)—would be approximately 0.995. And if, after 100,000 dice are rolled, 1001 roll three 6s in a row then—since that is approximately the average result if roughly one in 100,000 dice were biased—we would alter our value of P(HarryIsBiased) to be 0.00001. In that case, P(LauraIsBiased) ≈ 0.0022 rather than 0.686.

In short, if one is uncertain what proportion of dice are biased, but nevertheless has access to the actual results of the dice rolls, those results should bear on one’s credence for what proportion of dice are biased. Kotzen is therefore wrong to say that the results of the dice rolls in the multiple dice scenario are irrelevant to the probability of Laura being biased. Note that, nevertheless, the *mere fact* that other dice were rolled is irrelevant, i.e. weak defeatism may be true but Kotzen could still be correct that strong defeatism is false.

This revised dice scenario is the better analogy to real world studies for it is not certain what proportion of studies are on factors which actually have a real connection. Thus, it follows that just as weak defeatism is true of the revised dice scenario, weak defeatism is true when studies are considered.

### The independence argument

Kotzen has other arguments against (both strong and weak) defeatism. One is the ‘independence argument’ [1, p. 160]: given that the other studies are truly independent of one another, the results of one study says nothing about how other studies might turn out. If they say nothing whatsoever, both weak and strong defeatism are false.

Kotzen’s worry can be grasped first by returning to the dice analogy. Instead of one vat, imagine that there are a million vats. Each vat is filled by a different dice creating machine. Dials on the machines determine how many biased dice each machine produces. The dials on the machines have been independently set. Imagine a die is randomly selected from the vats. Call it ‘Harry’. If it rolls three 6s, then it would create an ‘improved single die scenario’. But imagine that it is not rolled, and instead is put back in the vat. We then roll one die each from 100,000 randomly selected vats. Of those dice which roll three 6s, one is selected. Call it ‘Laura’. This is the ‘improved multiple dice scenario’.

The results of the other 99,999 dice are prima facie irrelevant to the probability of Laura being biased. Imagine that all 99,999 dice rolled three 6s. In that case, as long as the dial settings were fixed independently of one another, the scenario nevertheless fails to tell us anything about the bias proportion *of the vat that Laura was pulled from*. The improved multiple dice scenario is a better analogy of the multiple study scenario: pairs of factors are analogous to the vats; each die is analogous to a study one might conduct on the pair; that the dials are independently fixed is analogous to the different studies being independent of one another, etc. So, by that analogy, what the results of the other studies are in the multiple study scenario seems irrelevant, i.e. weak defeatism is false.

But the devil is in the detail. P(LauraIsBiased) is a function of P(HarryIsBiased). Even in the improved multiple dice scenario, one might think that the results of the dice affect P(HarryIsBiased). Imagine that we have no idea of how to estimate P(HarryIsBiased). But imagine that prior to picking out Harry (and prior to rolling 100,000 dice, and prior to picking out Laura, etc.), we are allowed to roll some dice from the vats. Call them the ‘anterior dice’. The anterior dice are randomly selected such that each die may or may not have come from one of the vats from which we later draw one of the 100,000 dice and, indeed, we do not know whether some, or all, of the anterior dice came from the same vat. If we are genuinely at a loss as to how to estimate P(HarryIsBiased) then we would believe that it is equiprobable which vat any given die came from. In that case, we should fix P(HarryIsBiased) at a value mirroring the proportion of anterior dice which rolled three 6s. Then, just as clearly, if we roll 100,000 dice from amongst the vats, then the results of those dice are likewise going to update what we believe about P(HarryIsBiased). For instance, imagine that we rolled 10 anterior dice and estimated P(HarryIsBiased) on the back of that. That is not as good as rolling 100,009 which would help us better estimate that value. In the improved multiple dice scenario, the 99,999 rolls of the other dice are just as good as rolling extra anterior dice. The conclusion is that if one used the anterior dice method to estimate P(HarryIsBiased), then, clearly, the results of the other dice rolls are relevant to P(LauraIsBiased). That things are the same as they were in the multiple dice scenario should seem intuitive. In the improved multiple dice scenario, 100,000 dice are rolled from randomly selected vats. Imagine that the selected dice are put in a bucket before being rolled. This would now be a situation identical to the multiple dice scenario.

### Strained analogies?

Having added the use of the anterior dice process to the improved multiple dice scenario, there is now yet another variant scenario: the ‘anterior dice scenario’. If the anterior dice scenario were analogous to real world scenarios involving multiple independent studies, then weak defeatism would be false. However, if there were no analogy of the anterior dice process, our objection to Kotzen would not work. For there to be an analogy, there must be an *anterior studies process* whereby the probability of a study showing a real connection is, at least in part, based upon the proportion of studies thus far seen which demonstrate significant results. In the rest of this section, we argue that, in at least some cases, an anterior study process is used (and, further, *should* be used in at least some cases).

Before turning to a real world example, consider a fictional case. Imagine a researcher who has no expertise in, or knowledge relevant to, the field of peanut butter or cholesterol levels. Whilst she is perfectly rational, she has no idea how to estimate P(RealConnection), i.e. she has no idea what the prior probability is of peanut butter ingestion causing lower cholesterol. Nor does she have access to studies that are not independent of the peanut butter study. But she *does* have access to 1000 independent studies. Of those 1000 studies, 10 studies were conducted on factors that turned out to have a real connection. With nothing better to go on, the researcher should rationally estimate P(RealConnection) to be 0.01.

Now, imagine that this researcher reads an extra nine thousand studies. Were that to show that the proportion of studies conducted on factors with a real connection was, e.g., higher than the proportion in the next nine thousand, this should change her estimation. After reading 10,000 studies, if she saw that the proportion of studies conducted on factors with a real connection was in fact 1.3%, then she should correspondingly adjust P(RealConnection) to be 0.013. This is simply her deploying the anterior study process. In the multiple study scenario, were the researcher to discover the results of the other studies, then that would be yet more information to feed into a revision of P(RealConnection). Hence, the results of the multiple studies *do* affect the value of P(RealConnection)—and, therefore, the value of P(RealConnection|SignificantResults)—in the multiple study scenario (as compared to the single study scenario).

Nor is this relevant only to fiction for there are suitably similar real world cases. One example concerns diagnostic tests for diseases. Imagine someone tests positive for a disease. The subjective probability of the subject having the disease depends upon two things. First, the accuracy of the test: imagine it generates false positives 1% of the time (in uninfected subjects) and never generates false negatives. Second, the prevalence of the disease in the population: imagine that 1% of the population is infected. In such a case, the subjective probability of the subject having the disease would be (roughly) 0.5. And in this case, the subject would be a ‘study of one’ (with their positive test result being analogous to a die which has rolled three 6s). When one learns more about independently conducted studies (that is, learns more about the prevalence in the population), one’s subjective probability of the subject having the disease should vary. For instance, if one came to believe that it was not 1% of the population which was infected but 2%, then one’s subjective probability that the subject has the disease should now be roughly 0.66.

A second example involves testing DNA data. James Scott and James Berger use a Bayesian statistical model for testing DNA data [2]. Where *x*_*i*_ is the measured under- or over-expression of genes, and μ_*i*_ is *x*_*i*_’s true mean, it is natural to believe that (i) the prior probability that μ_*i*_ = 0 for any given *i* is (unless we know any better!) the same for every *i*, and (ii) that each value of μ_*i*_ is independent. Examining the different genes is thus equivalent to conducting independent multiple studies on whether different genes over or under express. And when it comes to fixing the prior probability of μ_*i*_ = 0 for any given *i*, Scott and Berger say that the ‘emphasis … is on letting the data themselves (i.e., the results of the independent tests on the genes)’ fix that prior probability [2, p. 2145]. Clearly, this is just the anterior studies process in action.

So, the anterior studies process *is* used in the real world. Moreover, were weak defeatism false, then these cases would have to be conducted differently (which—especially in the case of diagnostic testing—would be quite a surprise!). Since the analogy holds, Kotzen’s independence argument does not work.

## The generality argument

The final of Kotzen’s arguments which we consider in-depth is his ‘generality argument’: if defeatism is true, and the existence of some independent studies influence P(RealConnection|SignificantResults), it seems impossible to draw the line between which studies are relevant and which are not. Are studies about statins and lower cholesterol relevant? Or eating peanut butter and complications arising from heart bypass surgery? Or heart bypass surgery and memory loss (which is, *prima facie*, totally irrelevant!)? With no good answer to that question, the defeatist is in trouble [1, pp. 159–160]. One can also consider this objection in relation to diagnostic disease testing. If testing, say, for the presence of HIV, one might pay close attention to the results of other HIV tests to feed into one’s prior probabilities. But one need not pay such attention to any old independent study whatsoever. For instance, one would not pay such attention to the results of tests on whether people have chlamydia or diabetes.

We do not believe that this argument demonstrates that weak defeatism is false. Return to the drudgery of the fictional researcher wading through one thousand, and then ten thousand, independent studies in order to estimate a value of P(RealConnection). The researcher could be asked why she selected *those* studies. Should the ten thousand studies include studies about statins and lower cholesterol, or about peanut butter and heart bypass surgery complications, or about heart bypass surgery and memory loss, and so on? If the researcher is genuinely ignorant of medical issues, we believe that *all* such studies are relevant to that researcher. After all, what else should she do? Since there is no alternative to the one we suggest, we think it is obvious that one should use weak defeatist reasoning in this case—and that, therefore, Kotzen’s argument cannot be sound.

When someone is less ignorant than this imaginary researcher, it gets trickier. Improved information changes which studies are relevant. Returning to the anterior dice scenario, a set of dice, Π, is constructed. The results of those dice inform our prior probability of Harry being biased. If Harry could have come from any vat, and the dice which are members of Π could have come from any vat, the results of dice from Π should inform that probability. But if it were known that Harry came from amongst the first 50,000 vats, and if it were known which anterior dice came from vat numbers 1–50,000 and which came from vat numbers 50,001–100,000, then one would know only to include dice of the former type in Π on the grounds that the other dice are not saliently similar to Harry. The same thinking applies in the case of studies. Let *s* be a study which our researcher has read; let Σ be the set of studies intended for use in the anterior studies process when estimating the (prior) probability of a real connection between the factors involved in *s*; let *p* be the principle that our researcher used for selecting *s* in the first place. Similar to the dice case, every study in Σ should be selectable by principle *p*. If the researcher knows that *p* could never have selected a given study, then that study should not be included in Σ.

Problematically, there will be many such principles. Some principles appear to be apposite whilst others do not. For instance, in diagnostic testing, the results of other HIV tests seem pertinent to the probability of a given individual having HIV; thus a principle selecting all and only HIV tests seems apposite. In the case of the peanut butter study, a principle selecting all studies on lower cholesterol caused by a variety of common edible substances is likewise apposite. But there are principles which are *prima facie* odd and bizarre. For instance, imagine that a researcher came across the peanut butter study by accident whilst rummaging under their sofa. In a sense, the researcher has used a principle for reading studies which selects studies left under sofas. But it would be bizarre for Σ to have as members all and only those studies that have been discarded under sofas. This problem can be showcased by a further example. Imagine one reads the peanut butter study in the *Journal of Peanut Butter Studies*. Should Σ include all studies in that journal? Why should it not include all studies that *could* have appeared in that journal (so include, e.g., studies rejected by that journal but accepted elsewhere)? What if every morning I randomly select a journal to read and happened, that morning, to select *Journal of Peanut Butter Studies*—should Σ now contain studies from any journal whatsoever? There are a plethora of candidate principles, each generating a distinct set of studies for the anterior studies process where those studies are all—in some sense or another—similar to *s*. In light of this, Kotzen may renew his objection, saying that unless one can develop a hard and fast rule for picking out which principle is apposite, weak defeatism is scuppered.

We cannot develop such a hard and fast rule, but we do not believe this to be damning to weak defeatism. Σ is a set of studies saliently similar to *s* with regards to how *s* came to be read. Pinning down ‘salient similarity’ has proven difficult in other areas so it is no surprise that it proves difficult here. But in the same way that, in other areas, guidelines can be offered, some rough guidelines can be offered here. The idea that some resemblances are ‘genuine resemblances’ which better respect cuts in nature’s joints can be extended [3]. For instance, the first detected electron and the Eiffel Tower resemble one another in some respect (e.g. they resemble one another insofar as both of them are the only things mentioned in this sentence). But that does not mean they *genuinely* resemble each other. Some predicates are more natural than others. For instance, ‘__ is a negatively charged particle’ is a more natural predicate than ‘__ is mentioned in sentence __’ or ‘__ is an artefact designed by Gustave Eiffel’ [4, 5]. The first predicate ‘better cuts nature at the joints’ than the latter two. Things resemble one another to the extent that they fall under the same predicates; things genuinely resemble one another to the extent that they fall under the same natural predicates. Thus, the Eiffel Tower and an electron’s resemblance can be accounted for whilst simultaneously capturing the fact that they do not *genuinely* resemble each other.

This in place, one can get a sense of which principles are apposite. A principle for constructing sets of studies is better than another principle when it respects these genuine resemblances. That is, if principle *p*_1_ selects studies whose subject matter genuinely resembles *s* to one degree whilst principle *p*_2_ selects studies whose subject matter genuinely resembles *s* to a greater degree, one should opt for *p*_2_ over and above *p*_1_. The apposite principle is simply the principle that is to be preferred over all others. For example, HIV tests all concern themselves with the presence, or not, of a particular disease. Having a disease or not is a fairly natural resemblance. So, a principle selecting other HIV tests (and ignoring tests on other diseases) has a subject matter (i.e., people having HIV) that better genuinely resembles the original test than if tests on other diseases were also included. No wonder, then, that one would focus on a set of such tests when it comes to the anterior studies process. If, in the peanut butter study case, studies on edible substances and heart disease were selected, those studies would have subject matters more closely genuinely resembling that of the peanut butter study than if a principle selecting studies found under sofas were used. If the latter principle were used, one should probably believe that the set one constructed would contain studies with disparate subject matters—one should, therefore, favour using the former principle.

Thus, we suggest that—when faced with a plethora of principles—one should select the principle which, to the best of one’s knowledge, is best suited to constructing a set of studies with genuinely similar subject matters. Hence, one should change which sets are pertinent to the anterior studies process as one learns more about the world. The thoroughly ignorant researcher imagined above, who ploughs through ten thousand random studies, does not know that, say, peanut butter and lower cholesterol do not genuinely resemble the factors appearing in those ten thousand studies. Hence, she is justified in including them in her set for use in the anterior studies process. However, in contemporary diagnostic testing, we know that an infection of HIV genuinely resembles other infections of HIV more than it genuinely resembles an infection of chlamydia. Hence, the set includes the independent HIV tests, though not other studies or tests, and is the desirable set to inform the relevant prior probabilities. The more one learns about the world, the more one is clued in as to which sets are better suited to help estimate prior probabilities. Thus, there is a constraint on how sets of anterior studies are to be constructed.

The constraint is only rough. One could expand upon it by adding in further constraints. For instance, the resemblance of the subject matter of studies is just one desideratum. Another desideratum would be that an apposite principle would include as many studies as practically possible. Without that desideratum, one would always favour the set consisting of just that thing which most genuinely resembles its subject matter, i.e. *s* itself. But that would be totally uninformative. Hence, with informativeness in mind, one can build sets with multiple studies in them even though those studies imperfectly genuinely resemble the subject matter of *s*—one must balance the demand for informativeness with the demands concerning the genuine resemblance of subject matter.

A further constraint can be suggested. Studies should only be included in Σ if the prior probabilities of there being real connections between the factors in the studies are approximately the same before the results of any of the studies are examined. For instance, if the prior probability of peanut butter lowering cholesterol is 0.01 and the prior probability of peanut butter causing heart arrhythmia is 0.01, then both may be included in Σ. In the example case, one would include all (saliently similar) studies where it is believed that the prior probability of the factors having a real connection is 0.01. When the results of those studies are discovered, one gets more information on what proportion of those studies actually demonstrate a real connection. For instance, one might discover that of those studies one selected to be included in the anterior set, 2% demonstrated a real connection rather than, as was assumed, 1%. Information about the anterior studies is simply information about how good one is at estimating those prior probabilities. In the example case, one would discover that when the probability of factors having a real connection is estimated to be 0.01, this is generally an underestimate for studies included in Σ.

More could be said about the construction of anterior sets. This sketch of some constraints on the sets nevertheless ameliorates Kotzen’s worries. The original worry was that there are not any guidelines or restrictions on what studies count towards affecting one’s credences. The sketch shows that there *can* be principled reasons to delimit the sets of studies in some way. Whilst Kotzen thinks weak defeatism leads to absurd conclusions (e.g., that it might lead to the diagnostic testing of one disease taking into account the results of *prima facie* irrelevant diagnostic tests for other diseases), the sketch shows how to resist that line of reasoning. It also explains why our totally ignorant researcher is justified in examining all studies that she comes across. The constraints placed on the construction of the anterior set depend upon the knowledge that one has. The more knowledge one acquires, the greater the limits placed on the composition of the anterior set. In a case of a thoroughly ignorant researcher, no limits are placed on them and thus, when totally ignorant, every study *is* relevant to estimating one’s prior probabilities.

## Conclusion

This concludes our examination of the pertinent arguments Kotzen offers against weak defeatism.^2^ We now end by discussing how what we said bears on current medical practice.

First, we should note that explicitly deploying weak defeatist reasoning is not new. Not only are there the examples of diagnostic testing, and analysis of DNA data from Scott and Berger, but there are others as well. In one example, Jonathan Sterne and George Davey Smith have already noted much of what we have written about in this article, albeit in the context of interpreting *p* values [6]. A *p* value is the probability that either the observed result, or something more extreme than the observed result, would be observed were a real connection between studied factors not present. When it comes to interpreting *p* values, they are explicit that the results of independently conducted studies should guide one to the proportion of false alarms that one should presume there to be, which factors into informing the prior probability of there being a real connection between studied factors.

In a second example, the prior probability of a genetic variant being associated with a disease varies depending upon the number of functional genetic variants along with the number of variants which contribute to the disease. Data about the latter information (i.e., number of functional genetic variants and number of contributing variants) bears on the prior probability of association with a disease [7–9].

In a third example, John Ioannidis believes that the proportion of significant results appearing in a set of studies provides guidance as to the prior probability of there being real connections when significant results appear [10]. As an example, he imagines a whole genome association study testing 100,000 gene polymorphisms for association with schizophrenia [10, p. 699]. He is explicit that the prior probability of any given gene being so associated mirrors the proportion of genes that are associated. Again, this is simply the anterior studies process in action.

However, there are practical issues which make implementation of this weak defeatist reasoning tricky. For instance, virtually all medical studies are conducted using classical or frequentist, rather than Bayesian, methods [11]. The anterior study process, being Bayesian in nature, does not feature in works based on a classical methodology, scuppering attempts to use the anterior studies process to better inform one’s priors in the vast majority of studies.^3^

Further, the simplifying assumptions made of the peanut butter case paint a misleading picture of how easy it would be to build a statistical model involving the weak defeatist’s reasoning. For instance, along with Kotzen, we assumed that real connections are both an all or nothing affair and always turn up significant results when tested. In real world cases, where these assumptions do not apply, statistical modelling methods are more complex; therefore, the ratio of effort to reward for using weak defeatist reasoning may often be quite low. The same is true for other assumptions—for instance, we have ignored the influence of bias in studies.

Similarly, in many real world cases one often has *non*-*independent* studies available. Information from such studies is likely to swamp information garnered from independent studies when it comes to estimating prior probabilities. That is not to say that the information from the independent studies is irrelevant, but just that the modification made in light of it would, in most (but not all!) cases, likely make little difference. Given the probable small effect it would have, it will not, in many cases, be worth the effort to build the appropriate statistical model. In any case, it would be challenging to develop a statistical methodology to correctly synthesise independent and non-independent studies of different types.

But this is not to say that the reasoning of the weak defeatist is unsound—even if it turns out that in the real world, it is often less useful to pay attention to it. Moreover, whilst in many cases it is inefficient to build weak defeatist reasoning into a statistical model, that is not true of all cases—the above case of Scott and Berger’s analysis of DNA is one example in which the reasoning of the weak defeatist is not only pertinent but also economical to take into account.

## Footnotes

1 Kotzen recognises this disanalogy in [1, pp. 166, 177]; he says that it is incumbent upon the defeatist to explain why this disanalogy might be salient—the rest of this section explains just that.

2 Kotzen’s other arguments are irrelevant given the current dialectic. The *triviality problem* [1, p. 160]—that the defeatist appears to be weakening the evidence as a motivation for strong defeatism—is irrelevant as we do not deploy any such reasoning in arguing for weak defeatism. Similarly, the *psychological problem*—that defeatism is committed to thinking that the ‘private psychological states of researchers’ [1, p. 161] are relevant—is *prima facie* not a problem for, clearly, weak defeatism commits to no such thing. The *commutativity of evidence issue* [1, pp. 161–162]—that it is irrelevant which order one receives information as to what we draw from that information—is irrelevant as nothing we say indicates that the order is relevant.

3 Of course, this is merely a descriptive fact—just because most studies do not use Bayesian reasoning does not mean that they *should not* [6, 12].
